# Investigation and Characterization of Plasma-Treated Poly(3-hydroxybutyrate) and Poly(3-hydroxybutyrate-*co*-3-hydroxyvalerate) Biopolymers for an In Vitro Cellular Study of Mouse Adipose-Derived Stem Cells

**DOI:** 10.3390/polym10040355

**Published:** 2018-03-22

**Authors:** Chih-Kai Chang, Hui-Min David Wang, John Chi-Wei Lan

**Affiliations:** 1 Biorefinery and Bioprocess Engineering Laboratory, Department of Chemical Engineering and Materials Science, Yuan Ze University, No. 135, Yuan-Tung Road, Chungli, Taoyuan 320, Taiwan; kevinvecent@gmail.com; 2 Graduate Institute of Biomedical Engineering, National Chung Hsing University, No. 145, Xing-Da Road, South District, Taichung 402, Taiwan; davidw@dragon.nchu.edu.tw

**Keywords:** biocompatibility, biopolymers, hydrophilicity, hydrophobicity, in vitro cellular study, plasma surface modification, poly(3-hydroxybutyrate), poly(3-hydroxybutyrate-*co*-3-hydroxyvalerate)

## Abstract

Polyhydroxyalkanoates (PHAs) are a type of thermoprocessable and biodegradable polyester, which represent a potential sustainable replacement for fossil-fuel synthetic polymers, such as polypropylene and polyethylene. In recent years, copolymers of PHAs, i.e., poly(3-hydroxybutyrate) (PHB) and poly(3-hydroxybutyrate-*co*-3-hydroxyvalerate) (PHBV), have received attention for medical and packaging industrial applications, due to their biodegradable, toxic-free, and biocompatible nature. This study investigated and characterized plasma-treated PHB and PHBV films fermented with *Ralstonia eutropha* H16. The X-ray photoelectron spectroscopy (XPS) and water contact angle analyses on the plasma-treated PHB and PHBV film surfaces revealed an increase in the number of functional groups and contact angle degree, respectively, compared to that of the untreated films. In addition, an in vitro experiment of mouse adipose-derived stem cells showed better growth and adhesion of the cells on the surface of plasma-treated PHBV film. Overall, these results reveal that plasma surface modifications are useful in biomaterial development.

## 1. Introduction

There is an increasing demand to develop a new generation of materials either by synthetic or natural manner. These materials should be characterized with eco-friendly, biocompatible, or biodegradable properties [1,2]. Biopolymers, synthesized by living organisms, can meet such demands as well as maintain compatible physical, mechanical, and chemical properties to those conventional polymer materials. As the consequence, biopolymers represent an attractive value for industrial applications as packaging and biomedical materials. For instance, biopolymers can be conducted in the fields of tissue engineering, drug delivery, nano-medicine, and biomedical devices due to the several advantages, such as biodegradable property and non-cytotoxic effect [3,4,5,6,7].

PHAs are a large family of linear polyesters that are produced microbiologically in nature. Many microorganisms, such as bacteria, produce PHAs as intracellular carbon and energy storage reserves [3,5,6]. Poly(3-hydroxybutyrate) (PHB), the simplest polyester in the group of PHAs, is well documented and has been studied comprehensively. At present, poly(3-hydroxybutyrate-*co*-3-hydroxyvalerate) (PHBV), i.e., a PHA copolymer, which comprises of 3-hydroxybutyrate (3-HB) and 3-hydroxyvalerate (3-HV), is gaining interest in medical and packaging industrial applications. This is because PHBV is less crystalline and more flexible compared to PHB, and therefore more readily processed. However, being a hydrophobic polyester, the hydrophilicity properties of PHBV must be enhanced to increase its medical and packaging industrial applications potential [4,8,9,10].

In recent years, plasma treatment has been incorporated with biomaterials, such as biopolymers, to improve hydrophobicity, hydrophilicity, and biocompatibility [11,12,13]. Improved cell attachment and growth characteristics, as well as formation of new functional groups on the surface of the biomaterials can be achieved with plasma treatment [14]. For example, Wang et al. [4] studied the use of O_2_ and N_2_ plasma treatment on commercialized PHBV film, and revealed a strong improvement in hydrophilicity. Microwave ammonia plasma surface modification has been applied to PHB film and studied for a tissue engineering scaffold application [15]. In addition, plasma treatment has been applied to surface modifications of poly(ε-caprolactone) [16,17,18] and poly(lactic acid) [19,20,21,22,23].

The objective of this study was to improve the surface properties of PHB and PHBV films by increasing hydrophobicity, hydrophilicity, and compatibility using plasma treatments of 1,1,1,2-tetrafluoroethane (C_2_H_2_F_4_) and methane/oxygen (CH_4_/O_2_). In this study, both PHB and PHBV were produced through *Ralstonia eutropha* H16 fermentation. This was followed by analyses of X-ray photoelectron spectroscopy (XPS), water contact angle, and in vitro cellular mouse adipose-derived stem cells (ASCs) on the surface of the plasma-treated PHB and PHBV films. The results obtained were compared to those of untreated films.

## 2. Materials and Methods

### 2.1. Materials

PHB was produced through fermentation of culture medium containing glycerol (as the carbon source) and *R. eutropha* H16. Similarly, PHBV with 10 mol % of 3-HV was produced using the same procedure, but sodium propionate was added to the culture medium. All solvents and chemicals used were of analytical grade.

### 2.2. Fabrication of the PHB and PHBV Films Using Solvent Casting

The PHB and PHBV films (both top and bottom surfaces) were fabricated by solvent casting. The sample was dissolved in 5% (*w*/*w*) chloroform (Merck, Darmstadt, Germany; purity 99.9%) in a glass dish at room temperature until the chloroform evaporated completely. The PHB and PHBV films were then dried in a vacuum for 2 days and stored in a desiccator at room temperature until use.

### 2.3. Plasma Surface Modification of the PHB and PHBV Films

A gaseous plasma treatment using 1,1,1,2-tetrafluoroethane (C_2_H_2_F_4_) and methane/oxygen (CH_4_/O_2_) was carried out on the PHB and PHBV films in a home built cylindrical radiofrequency (RF) glow-discharge reactor (diameter: 10 cm; length: 22 cm). A preliminary test was conducted to assess the optimum working pressure, RF power, and the standard gas plasma treatment procedure involving evacuation of the chamber. Then, the reactor was filled with C_2_H_2_F_4_ and CH_4_/O_2_ at working pressures of 800 and 120 mTorr, respectively. A 20 W RF generator (frequency: 13.56 MHz) was activated for 10 and 1 min for C_2_H_2_F_4_ and CH_4_/O_2_, respectively. The sample was removed from the reactor after 10–15 min of complexion of the gas plasma treatment and stored under vacuum until use (≤3 months).

### 2.4. Analytical Procedures

#### 2.4.1. Nuclear Magnetic Resonance Spectroscopic Analysis

**Figure 1:**
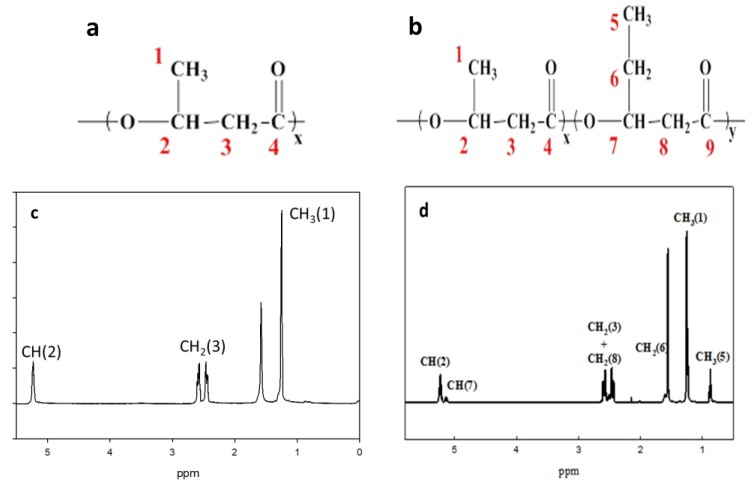
(**a**) PHB monomer structure; (**b**) PHBV monomer structure; (**c**) ^1^H NMR spectrum of PHB copolymer in CDCl_3_ solution; (**d**) ^1^H NMR spectrum of PHBV copolymer in CDCl_3_ solution.

The solution ^1^H nuclear magnetic resonance (NMR) spectrum was recorded on an Ultrashield 500 MHz NMR spectrometer (Bruker, Karlsruhe, Germany) at room temperature using CDCl_3_ as the solvent. The ^1^H NMR spectra obtained for the PHB and PHBV films were used to estimate monomer unit content. The molar fraction of the 3-HB unit of the fractionated PHB and PHBV films was estimated from the relative integrated CH_2_ (3) resonance, as shown in Figure 1a,b, respectively. In addition, the molar fraction of the 3-HV unit of the fractionated PHBV film was estimated from the relative integrated CH_3_ (5) resonance (Figure 1b).

#### 2.4.2. Water Contact Angle Measurements

The surface wettability of the plasma-treated and untreated PHB and PHBV films was measured according to the sessile drop method using a goniometer (Model 100SB Sintateck, Taipei, Taiwan). In brief, 1 μL of distilled water was dropped on the surface of different substrates and was allowed to stabilize for 10 s before measuring the water contact angle of the sample. The drop image was subsequently stored. The measurement of the static contact angle was fully automated using a charge-coupled device (CCD) video camera with the computer software provided with the instrument. An average value was obtained through five measurements of the contact angle value for each sample.

#### 2.4.3. X-ray Photoelectron Spectroscopy Analysis

The surface elemental composition and atomic concentration of the plasma-treated and untreated PHB and PHBV films were analyzed using X-ray photoelectron spectroscopy (XPS) (K-Alpha X-ray photoelectron spectrometer system; Thermo Fisher Scientific, Waltham, MA, USA). The analysis of the spectra of these films was done using peak fitting and the XPS system data processing unit.

#### 2.4.4. Surface Morphology Study

The surface morphology of the plasma-treated and untreated PHB and PHBV films was characterized using scanning electron microscopy (SEM) (JSM-5600; Hitachi, Tokyo, Japan). These films were gold-coated before loading into the SEM chamber with a 10 kV acceleration voltage.

#### 2.4.5. Preliminary In Vitro Cultured Mouse ASCs Study

The cellular properties of the plasma-treated and untreated PHB and PHBV films was studied using preliminary in vitro cultured mouse ASCs as described previously with slight modifications [24,25,26,27]. A cell suspension of 10^4^ cells/mL was first prepared before seeding. Triplicate specimens of each sample were sterilized in 70% (*v*/*v*) ethanol and were washed in culture medium before proceeding to the cell culture procedure. The cells (5 mL) were placed in a multi-well tissue culture polystyrene plate, including a negative control. The cells were observed for 48 h in a CO_2_ controlled incubator at 37 °C. Then, the cell suspension was washed with phosphate buffered saline (PBS) solution, and the cells were stained using a PKH26 Red Fluorescent Cell Linker Kit (Sigma-Aldrich, St. Louis, MO, USA). A fluorescence microscope (ZEISS, Oberkochen, Germany) was used to observe the morphology of the stained cells that adhered to the substrate. Moreover, we also performed a longer cell culturing period with 7 days to verify the cellular proliferation of mouse ASCs (MTT assay).

#### 2.4.6. Cell Viability Using MTT Assay

The effect of viability of mouse ASCs cultured on the plasma-treated and untreated PHB and PHBV films was investigated using MTT assay as described previously with slight modifications [24,25,26]. The mouse ASCs were seeded at 1 × 10^5^ cells/well in a 96-well plate and were incubated overnight. The cells were dissolved in 10% dimethyl sulfoxide (DMSO) (medium was mixed with DMSO) and were then allowed to stand in 0.5 mg/mL 3-(4,5-dimethylthiazol-2-yl)-2,5-diphenyltetrazolium bromide (MTT) for 2 h. The crystals were dissolved in DMSO and subsequently shaken for 10 min in the dark. The absorbance value (OD) was measured at 595 nm with a microplate reader (UV–Vis; BioTek, Winooski, VT, USA). Cell viability (%) was calculated using Equation (1).

(1) $$\mathrm{Cell}\;\mathrm{viability}\;\left(\%\right)=\;\frac{{\mathrm{OD}}_{\mathrm{sample}}}{{\mathrm{OD}}_{\mathrm{control}}}\;\times 100$$

#### 2.4.7. Study of Angiogenic Growth Factors

Two angiogenic growth factors, i.e., transforming growth factor-beta (TGF-β) and vascular endothelial growth factor (VEGF), secreted from mouse ASCs cultured on the plasma-treated and untreated PHB and PHBV films were determined by enzyme-linked immunosorbent assay (ELISA) (BlueGene, American Research Products Inc., Waltham, MA, USA).

#### 2.4.8. Western Blot Analysis

The western blot analysis was conducted as described previously with slight modifications [24]. A sample from a mouse ASC cultured on the plasma-treated and untreated PHB and PHBV films was lysed with RIPA lysis buffer. The sample proteins were separated using sodium dodecyl sulfate-polyacrylamide gel electrophoresis (SDS-PAGE) and were subsequently transferred from the gel onto a polyvinylidene difluoride (PVDF) membrane using an electric current. Then, the membrane was washed mildly with 1× TBST (mixture of tris-buffered saline (TBS) and Tween 20) to remove any traces of skim milk and put in a TBST box. The membrane was incubated with respective primary antibodies followed by secondary horseradish peroxidase-conjugated antibodies for 1 h. The protein signals were visualized using a polymerase chain reaction (PCR) system (Applied Biosystems StepOne Plus^TM^ Real-Time PCR System Thermal; Foster City, CA, USA).

### 2.5. Statistical Analysis

The statistical analysis was performed using SPSS statistical software (SPSS version 23.0 for window, IBM Corporation, Selangor, Malaysia). Triplicate readings were recorded and were analyzed statistically, and the values were expressed as mean ± of standard deviation (SD). The data were subjected to one-way ANOVA (analysis of variance), and the mean differences were compared using Tukey HSD post-hoc multiple comparisons test. The data were considered statistically significant difference where *p* < 0.05.

## 3. Results and Discussion

### 3.1. Nuclear Magnetic Resonance Spectral Analysis

The NMR spectral analysis was conducted to verify the chemical structures of PHB and PHBV, as shown in Figure 1a,b, respectively. Figure 1c,d depicts the ^1^H NMR spectra of the PHB and PHBV monomers in CDCl_3_, respectively. All signals were assigned to protons in the monomer repeating unit. As shown in Figure 1c (PHB), the methyl protons (–CH_3_ (1)) of the 3-HB side chain corresponded to the doublet at 1.3 ppm, whereas the presence of the methylene protons (–CH_2_ (3)) of the 3-HB main chain was indicated by two multiplets observed at approximately 2.5 ppm. The methines (–CH (2)) of the 3-HB bulk structure chain corresponded to two main multiplets, which were observed at about 5.2 ppm.

On the other hand, the ^1^H NMR spectrum of PHBV showed that the methyl protons (–CH_3_ (1)) of the 3-HB side chain corresponded to the doublet at 1.3 ppm, while the methyl protons (–CH_3_ (5)) of the 3-HV side chain was represented by the triplet at 0.9 ppm. The methylene protons (–CH_2_ (6)) of the 3-HV side chain corresponded to the multiplet at 1.6 ppm, and the presence of methylene protons (–CH_2_ (3 and 8)) of the 3-HB–3-HV main chains was indicated by two multiplets observed at about 2.5 ppm. The methines (–CH (2 and 7) of the 3-HB and 3-HV bulk structure chains corresponded to two multiplets, which were observed at about 5.2 ppm (Figure 1d). 

### 3.2. Water Contact Angle Analysis

**Figure 2:**
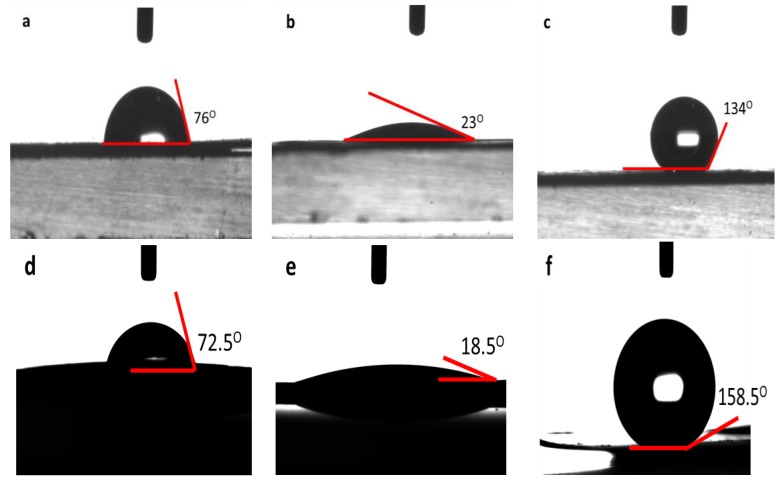
Water contact angle of (**a**) untreated (normal), (**b**) CH_4_/O_2_ plasma-treated (hydrophilic), (**c**) C_2_H_2_F_4_ plasma-treated (hydrophobic) PHB films and (**d**) untreated (normal), (**e**) CH_4_/O_2_ plasma-treated (hydrophilic), (**f**) C_2_H_2_F_4_ plasma-treated (hydrophobic) PHBV films.

The water contact angle analysis is a rapid, simple, and direct method to evaluate the hydrophobicity and hydrophilicity characters of a surface. Figure 2 shows the water contact angle of the plasma-treated and untreated PHB and PHBV films. Both top and bottom surfaces of the PHB and PHBV films were fabricated using solvent casting with chloroform, and both surfaces showed similar surface characteristics, as well as similar response on the wettability analysis and other test in this study. The untreated PHB and PHBV films recorded water contact angle values of 76.0° and 72.5°, respectively (Figure 2a,d). A decrease in the water contact angle was observed on the CH_4_/O_2_ plasma-treated PHB (from 76.0° to 23.0°) and PHBV (from 72.5° to 18.5°) films, as shown in Figure 2b,e, respectively. In contrast, the C_2_H_2_F_4_ plasma-treated PHB and PHBV films showed an increase water contact angle compared to that of the untreated films. An increase in the water contact angle from 76.0° to 134.0° was noted on the C_2_H_2_F_4_ plasma-treated PHB film (Figure 2c), while the C_2_H_2_F_4_ plasma-treated PHBV film showed an increase of water contact angle of 86.0° (from 72.5° to 158.5°) compared to that of the untreated film (Figure 2f).

A reduction in the water contact angle implies an increase in wettability (hydrophilicity) of the CH_4_/O_2_ plasma-treated PHB and PHBV films. In contrast, the increase in the water contact angle value clearly shows that the surface of the C_2_H_2_F_4_ plasma-treated PHB and PHBV films are more hydrophobic. The improved surface hydrophilicity of the PHB and PHBV films was probably due to the cleavage of hydrophobic groups and newly formed hydrophilic groups [4], whereas the increase in the hydrophobicity of the PHB and PHBV films could be due to the presence of fluorocarbons (CF_x_) that eliminate or reduce water adhesion on the surface [12]. These results were further confirmed using X-ray photoelectron spectroscopy analysis. 

### 3.3. X-ray Photoelectron Spectroscopy Analysis

**Figure 3:**
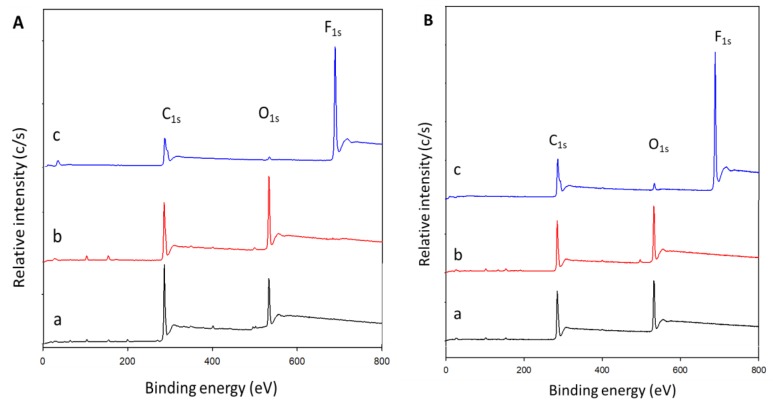
XPS spectral analysis on the surface of (**A**) PHB and (**B**) PHBV films. a, b, and c represent untreated, CH_4_/O_2_, and C_2_H_2_F_4_ plasma-treated, respectively.

The XPS analysis investigated the plasma-induced surface modifications on the PHB and PHBV films. The chemical environment of the atoms could change because of plasma surface modifications. This is largely due to cleavage or newly formed chemical bonds induced by the attachment of plasma particles that carry high energy onto the surface of the sample material [4]. Figure 3 shows the spectral analysis of the structure and the chemical states of the atoms on the surface of the CH_4_/O_2_ and C_2_H_2_F_4_ plasma-treated and untreated PHB and PHBV films. In addition, the calculated atomic percentages of each species of the plasma-treated (CH_4_/O_2_ and C_2_H_2_F_4_) and untreated PHB and PHBV films based on the spectral analysis are presented in Table 1.

The C_1s_ and O_1s_ peaks were expected to appear at binding energies of 285 and 532 eV, respectively, on the untreated PHB (Figure 3A(a)) and PHBV films (Figure 3B(a)). After the CH_4_/O_2_ plasma treatment, the O_1s_ peak for the PHB and PHBV films became stronger, as depicted in Figure 3A(b),B(b), respectively. Oxygen content increased from 21.00% to 30.02%, while carbon content dropped relatively after the CH_4_/O_2_ plasma surface modification on PHB film. Hence, the O/C ratio increased from 0.27 to 0.43. On the other hand, the CH_4_/O_2_ plasma-treated PHBV film showed an increase of 12.10% oxygen content and a relative reduction in carbon content. Thus, the O/C ratio increased from 0.37 to 0.64 (Table 1).

On the other hand, a F_1s_ peak appear at binding energy of 688 eV, became an evident on the existence of fluorine on both the C_2_H_2_F_4_ plasma-treated PHB and PHBV films (Figure 3A(c),B(c)). Our results reveal that fluorine, which represents a new functional group, was incorporated onto the surface of the PHB and PHBV films, with fluorine contents of 43.50% and 39.60%, respectively. The C_2_H_2_F_4_ plasma-treated PHB film showed reductions in oxygen and carbon contents to 1.70% and 54.80%, respectively, and thereby the O/C ratio decreased significantly to 0.03. Similarly, the oxygen and carbon contents of the C_2_H_2_F_4_ plasma-treated PHBV film decreased to 3.10% and 57.30%, respectively; hence, the relative O/C ratio also dropped (0.05) (Table 1).

### 3.4. Surface Morphology Analysis

**Figure 4:**
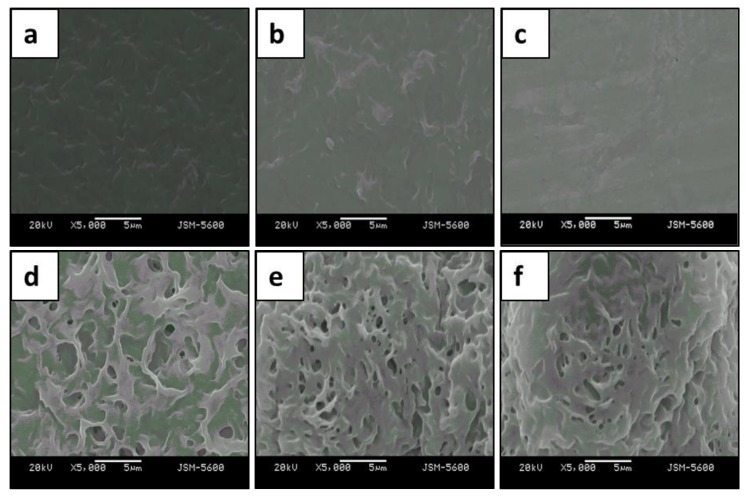
SEM of (**a**–**c**) PHB and (**d**–**f**) PHBV films. (**a**,**d**): normal; (**b**,**e**): hydrophilic; (**c**,**f**): hydrophobic.

The surface characteristics of the untreated (normal) and plasma-treated (hydrophilic and hydrophobic) PHB and PHBV films are shown in Figure 4. As mentioned in Section 3.2, both top and bottom surfaces of the PHB and PHBV films showed similar response because both surfaces possess similar surface characteristics. We detected a difference in the surface morphology between the plasma-treated and untreated PHB and PHBV films. The normal PHB and PHBV films showed a dense and low-porosity surface, whereas both the hydrophilic and hydrophobic PHB and PHBV films showed a line interleaving with a high porosity surface (Figure 4a–f). Plasma surface modifications that required high energy activation resulted in roughening of the membrane surface. In addition, a thin film was observed due to plasma deposition onto the substrate that increased surface density. In general, the plasma only affects a depth range of several hundred Å to 10 μm, and only the surface modification is activated without modifying the native properties of the material. The surface characteristics of the polymer are very crucial, as they greatly influence interactions between the host and implant. This is because their activity platform often takes place on the surface of the host. In short, plasma surface modifications roughened the membranes of both the treated PHB and PHBV films.

### 3.5. Preliminary In Vitro Cultured Mouse ASCs Study

**Figure 5:**
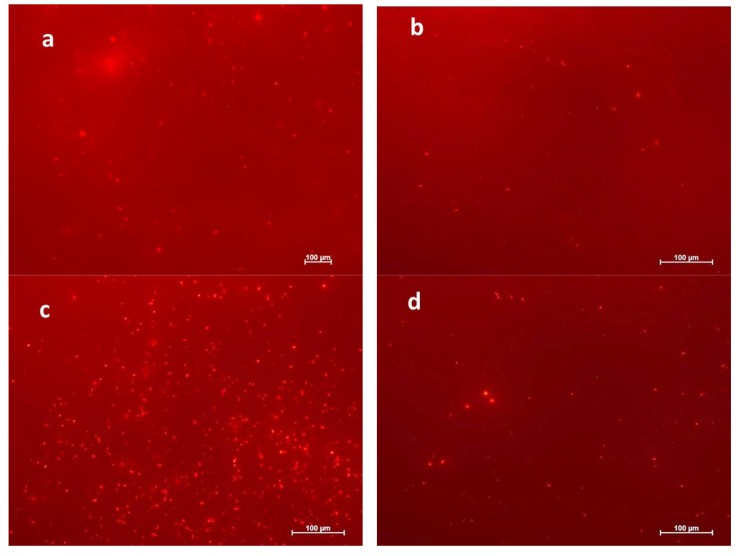
Preliminary results on cell adhesion of in vitro cultured mouse ASCs on (**a**) CH_4_/O_2_ plasma-treated (hydrophilic), (**b**) C_2_H_2_F_4_ plasma-treated (hydrophobic) PHB films and (**c**) CH_4_/O_2_ plasma-treated (hydrophilic), (**d**) C_2_H_2_F_4_ plasma-treated (hydrophobic) PHBV films.

The cellular behavior of a biomaterial is usually used to analyze the biocompatibility characteristics of the cell. The entire process of adhesion and spreading of cells consists of cell adhesion, growth of filopodia, cytoplasmic webbing, followed by flattening of the cell mass and ruffling of the peripheral cytoplasm, which progresses in a sequential fashion [28,29]. The attachment and growth of mouse ASCs were observed on the untreated (normal), CH_4_/O_2_ plasma (hydrophilic) and C_2_H_2_F_4_ plasma (hydrophobic)-treated PHB and PHBV films (Figure 5). Cell proliferation on the normal PHB and PHBV surfaces was negligible (data not shown). In contrast, improved attachment and growth of mouse ASCs were observed on both the hydrophilic and hydrophobic PHB and PHBV surfaces. The CH_4_/O_2_ plasma treatment (hydrophilic) provided a better result than its counterpart, i.e., C_2_H_2_F_4_ plasma treatment (hydrophobic) in terms of cell proliferation. Additionally, the hydrophilic and hydrophobic PHBV films showed better cell proliferation ability compared to that of the PHB films. These are preliminary results on in vitro cellular study of mouse ASCs, and the further responses on in vitro cultured mouse ASCs on the plasma-treated and untreated PHB and PHBV films were investigated using MTT assay, angiogenic growth factors study, and western blot analysis.

The surface properties of a biomaterial influence cell adhesion on the surface, which can be due to surface charge. In other words, the electrostatic interaction between the cell and the biomaterial surface has a crucial role in cell adhesion. A high negatively charged surface is an undesirable factor for cell growth [30]. Although the plasma-treated PHB and PHBV films have a similar molecular composition to the untreated PHB and PHBV films, the plasma treatment improved hydrophilicity and hydrophobicity, but decreased the zeta potential of the polymer surface. In addition, the plasma surface modifications roughened the surface membranes of both the treated PHB and PHBV films, which also influenced the cell adhesion. In short, plasma treatment influenced the surface wettability, surface roughness, and surface charge, in which could greatly influence cell attachment and growth. In other words, the plasma treatment induced better cell proliferation than that of the normal PHB and PHBV films. Moreover, our observations suggest that the plasma-treated PHBV film is well supported for cell growth and adhesion compared to that of the PHB film, indicating a potential application of plasma-treated PHBV film as a good biocompatible biomaterial.

### 3.6. Viability of Mouse ASCs Cultured on the PHB and PHBV Films

**Figure 6:**
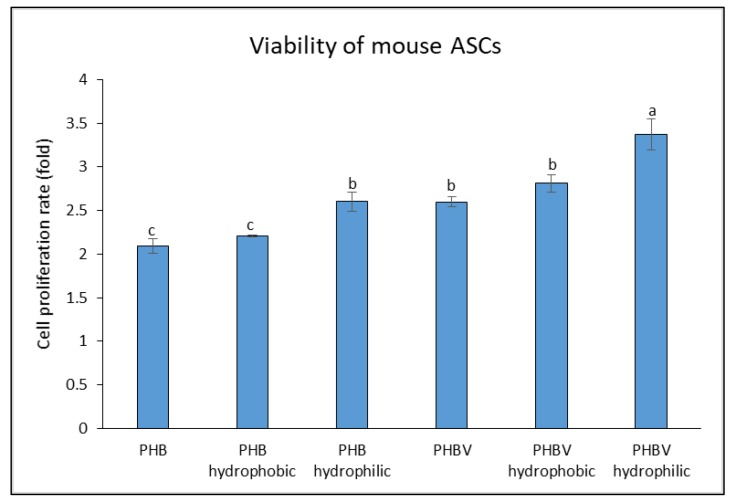
Cell proliferation rate of in vitro cultured mouse ASCs on PHB, PHB hydrophobic, PHB hydrophilic, PHBV, PHBV hydrophobic, and PHBV hydrophilic. Values are mean ± SD of triplicate readings. Different letter(s) represent a significant different (*p* < 0.05) using Tukey’s test.

Figure 6 shows the cell proliferation rate of in vitro cultured mouse ASCs on the plasma-treated and untreated PHB and PHBV films. The normal and plasma-treated (hydrophilic and hydrophobic) PHBV films showed higher rates of cell proliferation among the others. In particular, both hydrophilic PHB and PHBV showed the highest cell proliferation rates compared to their respective groups (i.e., normal and hydrophobic). However, hydrophilic PHBV resulted in the overall highest cell proliferation rate among the groups, indicating that it could be a good biomaterial for cell attachment and growth due to the presence of 3-HV, which results in a less crystalline and more flexible biomaterial. In addition, hydrophilic PHBV possessed the highest oxygen content of 39.20% (Table 1). The highest oxygen content is suggested to demonstrate the overall highest cell proliferation.

### 3.7. Study of Angiogenic Growth Factors

**Figure 7:**
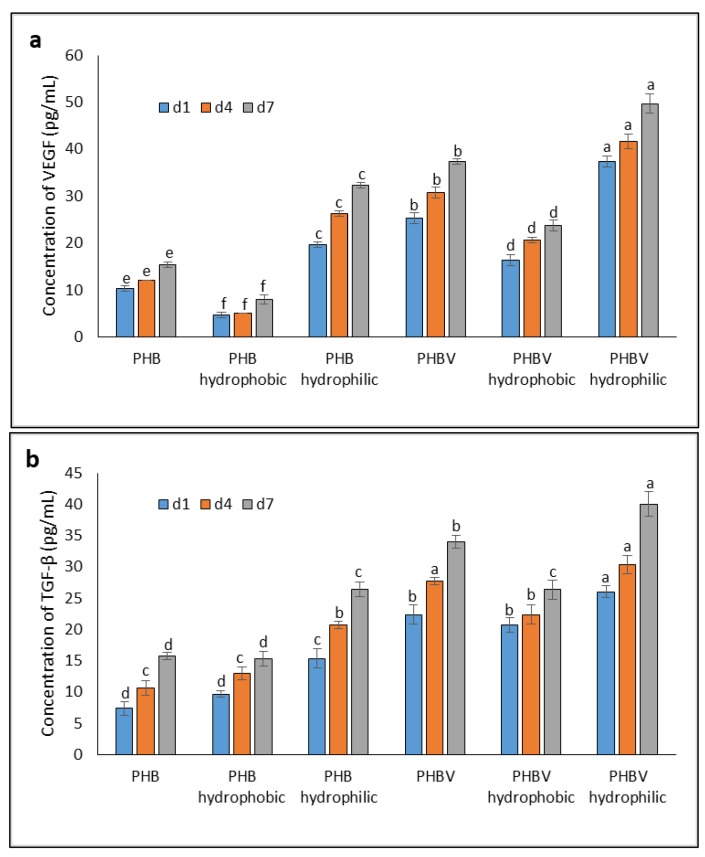
Concentrations of (**a**) VEGF (**b**) TGF-β secreted from in vitro cultured mouse ASCs on PHB and PHBV films over a 7-days period. Values are mean ± SD of triplicate readings. Different letter(s) represent a significant different (*p* < 0.05) using Tukey’s test within d1, d4, and d7 (d represents day).

A fast healing process of a wound is stimulated when there is rapid cell proliferation, and this could be influenced by biocompatibility of a biomaterial. Many growth factors, such as VEGF and TGF-β, stimulate wound regeneration [22]. TGF-β and VEGF are growth factors secreted by many cell types, such as platelets, macrophages, keratinocytes, fibroblasts, and ASCs [31]. Cell proliferation and the growth factors secreted by in vitro cultured mouse ASCs on the PHB and PHBV films (plasma-treated and untreated) were measured in days 1, 4, and 7, and the medium was changed every other day. In this experiment, the amount of VEGF and TGF-β secreted by mouse ASCs was analyzed, as shown in Figure 7a,b, respectively.

As depicted in Figure 7a, both the hydrophobic PHB and PHBV films showed the lowest concentrations of VEGF (i.e., 8.00 ± 1.00 and 23.67 ± 1.15 pg/mL, respectively) compared to their respective groups (i.e., untreated and hydrophobic PHB and PHBV), and the increase in VEGF concentration from days 1 to 7 was prominently delayed. In contrast, both the hydrophilic PHB and PHBV films recorded the highest concentrations of VEGF at 32.33 ± 0.58 and 49.67 ± 2.08 pg/mL, respectively, on day 7. Similarly, the TGF-β concentrations secreted from the PHB and PHBV films followed a similar trend as those of VEGF. The TGF-β concentrations on the hydrophilic PHB and PHBV films were highest at 26.33 ± 1.15 and 40.00 ± 2.00 pg/mL, respectively, on day 7 compared to their respective groups (i.e., untreated and hydrophobic PHB and PHBV) (Figure 7b). Overall, the hydrophilic PHBV film resulted in the highest concentrations of VEGF and TGF-β secreted by mouse ASCs among the others.

VEGF induces angiogenesis and extracellular matrix (ECM) precipitation in vivo. It can activate endothelial cells to promote cell proliferation and activate tyrosine kinase to promote angiogenesis. VEGF is secreted, expressed, and produced by several somatic cells, including keratinocytes, fibroblasts, neutrophils, macrophages, platelets, endothelial cells, and smooth muscle cells [24]. It facilitates ECM reconstruction around a wound. Similarly, TGF-β is a multifunctional protein that influences cell growth, differentiation, apoptosis, and immune regulation. The production of TGF-β is similar to the production of VEGF, and it is involved in wound contraction, angiogenesis, cell penetration, connective tissue regeneration, inflammation, as well as fibrotic scar formation and re-epithelialization [24,32]. Previous studies [32] have reported that TGF-β secreted from rat ASCs induces a higher expression of type I collagen and cell cycle regulatory proteins as well as enhances migration of fibroblasts. TGF-β allows fibroblasts and myofibroblasts to form a constrictive penetrative force, which aids in wound healing in the ECM. TGF-β upregulates the tissue inhibitor of metalloproteinase-1 gene and collagen production, while downregulates transcription of the matrix metalloproteinase-1 gene and protein secretion during ECM formation.

We discovered that VEGF and TGF-β concentrations were appreciably enhanced in mouse ASCs cultured on the PHB and PHBV films (with or without plasma treatment), suggesting that the augmented VEGF and TGF-β productions might be due to an increase in the production of platelet-derived growth factor. The MTT assay showed that the cells were not toxic after feeding *Bacillariophyceae* [22]. We used the MTT and ELISA methods to evaluate the proliferation rate and the quantities of TGF-β and VEGF secreted, respectively. The results reveal that there was an increase in VEGF and TGF-β secretion, indicating that *Bacillariophyceae* aided in wound repair [33].

### 3.8. Western Blot Analysis

**Figure 8:**
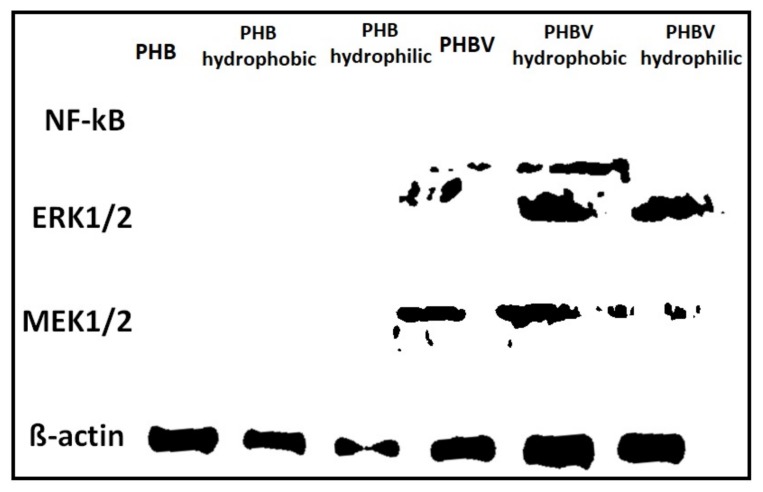
Protein expression level on plasma-treated and untreated PHB and PHBV films.

Figure 8 shows the results of a western blot analysis on the protein expression level for the plasma-treated and untreated PHB and PHBV films. We chose proteins involved in wound repair and cell proliferation to verity our hyopthesis. Our results reveal that the PHBV with and without plasma surface modifications showed enhanced levels of protein expression compared to those of PHB films, except nuclear factor-kappaB (NF-kB) protein expression. This finding suggests that PHBV exhibits better cell proliferation and wound repair functions compared to those of PHB, and its performance was greatly improved after the plasma surface modification. Thus, PHBV may serve as an attractive biomaterial for various industrial and medical applications [21,28].

## 4. Conclusions

This study reveals that plasma surface modifications improved the properties of biopolymers, such as PHB and PHBV, and avoided the use of organic solvents that can cause environmental hazards. The XPS and water contact angle analyses on the CH_4_/O_2_ and C_2_H_2_F_4_ plasma-treated PHB and PHBV films verified that the functional groups as well as hydrophilic and hydrophobic properties of the treated films were altered compared to those of untreated films. These results infer the importance of a detailed characterization of the surface of plasma-treated substrates designed as part of a strategy to optimize the surface properties of a specific cell biomaterial. In addition, in vitro cultured mouse ASCs on the plasma-treated and untreated PHB and PHBV films demonstrated better cell proliferation and wound repairing functions with the use of the plasma-treated and untreated PHBV films. Also, the plasma-treated PHBV film provided a better result compared to that of the untreated film. Overall, the results strongly suggest that plasma surface modifications can be useful for biomaterial development.

## Figures and Tables

**Table 1:** Atomic percentages of CH_4_/O_2_ and C_2_H_2_F_4_ plasma-treated and untreated PHB and PHBV films based on the spectral analysis.

Condition	PHB Film				PHBV Film			
	C (%)	O (%)	F (%)	O/C	C (%)	O (%)	F (%)	O/C
Untreated (Normal)	79.00	21.00	0.00	0.27	72.90	27.10	0.00	0.37
CH_4_/O_2_ plasma-treated (Hydrophilic)	69.98	30.02	0.00	0.43	60.80	39.20	0.00	0.64
C_2_H_2_F_4_ plasma-treated (Hydrophobic)	54.80	1.70	43.50	0.03	57.30	3.10	39.60	0.05

## Abbreviations

ASCs	adipose-derived stem cells
NMR	nuclear magnetic resonance
PHAs	polyhydroxyalkanoates
PHB	poly(3-hydroxybutyrate)
PHBV	poly(3-hydroxybutyrate-co-3-hydroxyvalerate)
SD	standard deviation
SEM	scanning electron microscope
TGF-β	transforming growth factor-beta; VEGF, vascular endothelial growth factor
XPS	X-ray photoelectron spectroscopy
3-HB	3-hydroxybutyrate
3-HV	3-hydroxyvalerate
